# Supplementary material for: Impact of salivary and pancreatic amylase gene copy numbers on diabetes, obesity, and functional profiles of microbiome in Northern Japanese population
Source: Sci Rep. 2022 May 10;12:7628. doi: 10.1038/s41598-022-11730-7 (PMC9090785; doi:10.1038/s41598-022-11730-7)
Supplement: Supplementary file 1 — Supplementary Information 1. [file 41598_2022_11730_MOESM1_ESM.pdf]

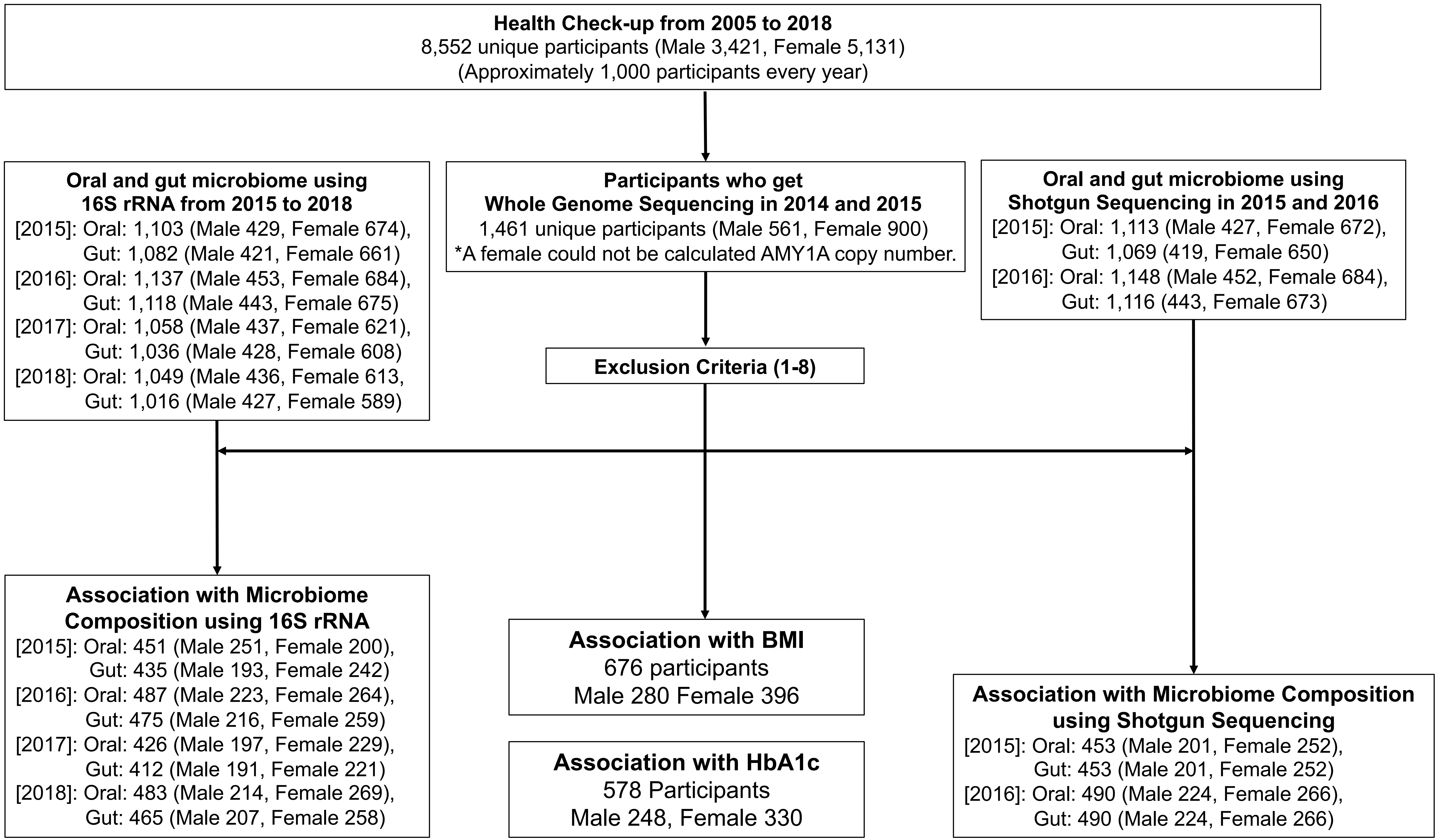

Health Check-up from 2005 to 2018
8,552 unique participants (Male 3,421, Female 5,131)
(Approximately 1,000 participants every year)
Oral and gut microbiome using 16S rRNA from 2015 to 2018
[2015]: Oral: 1,103 (Male 429, Female 674),
Gut: 1,082 (Male 421, Female 661)
[2016]: Oral: 1,137 (Male 453, Female 684),
Gut: 1,118 (Male 443, Female 675)
[2017]: Oral: 1,058 (Male 437, Female 621),
Gut: 1,036 (Male 428, Female 608)
[2018]: Oral: 1,049 (Male 436, Female 613,
Gut: 1,016 (Male 427, Female 589)
Participants who get Whole Genome Sequencing in 2014 and 2015
1,461 unique participants (Male 561, Female 900)
*A female could not be calculated AMY1A copy number.
Oral and gut microbiome using Shotgun Sequencing in 2015 and 2016
[2015]: Oral: 1,113 (Male 427, Female 672),
Gut: 1,069 (419, Female 650)
[2016]: Oral: 1,148 (Male 452, Female 684),
Gut: 1,116 (443, Female 673)
Exclusion Criteria (1-8)
Association with Microbiome Composition using 16S rRNA
[2015]: Oral: 451 (Male 251, Female 200),
Gut: 435 (Male 193, Female 242)
[2016]: Oral: 487 (Male 223, Female 264),
Gut: 475 (Male 216, Female 259)
[2017]: Oral: 426 (Male 197, Female 229),
Gut: 412 (Male 191, Female 221)
[2018]: Oral: 483 (Male 214, Female 269),
Gut: 465 (Male 207, Female 258)
Association with BMI
676 participants
Male 280 Female 396
Association with HbA1c
578 Participants
Male 248, Female 330
Association with Microbiome Composition using Shotgun Sequencing
[2015]: Oral: 453 (Male 201, Female 252),
Gut: 453 (Male 201, Female 252)
[2016]: Oral: 490 (Male 224, Female 266),
Gut: 490 (Male 224, Female 266)
