# Supplementary material for: Impact of salivary and pancreatic amylase gene copy numbers on diabetes, obesity, and functional profiles of microbiome in Northern Japanese population
Source: Sci Rep. 2022 May 10;12:7628. doi: 10.1038/s41598-022-11730-7 (PMC9090785; doi:10.1038/s41598-022-11730-7)

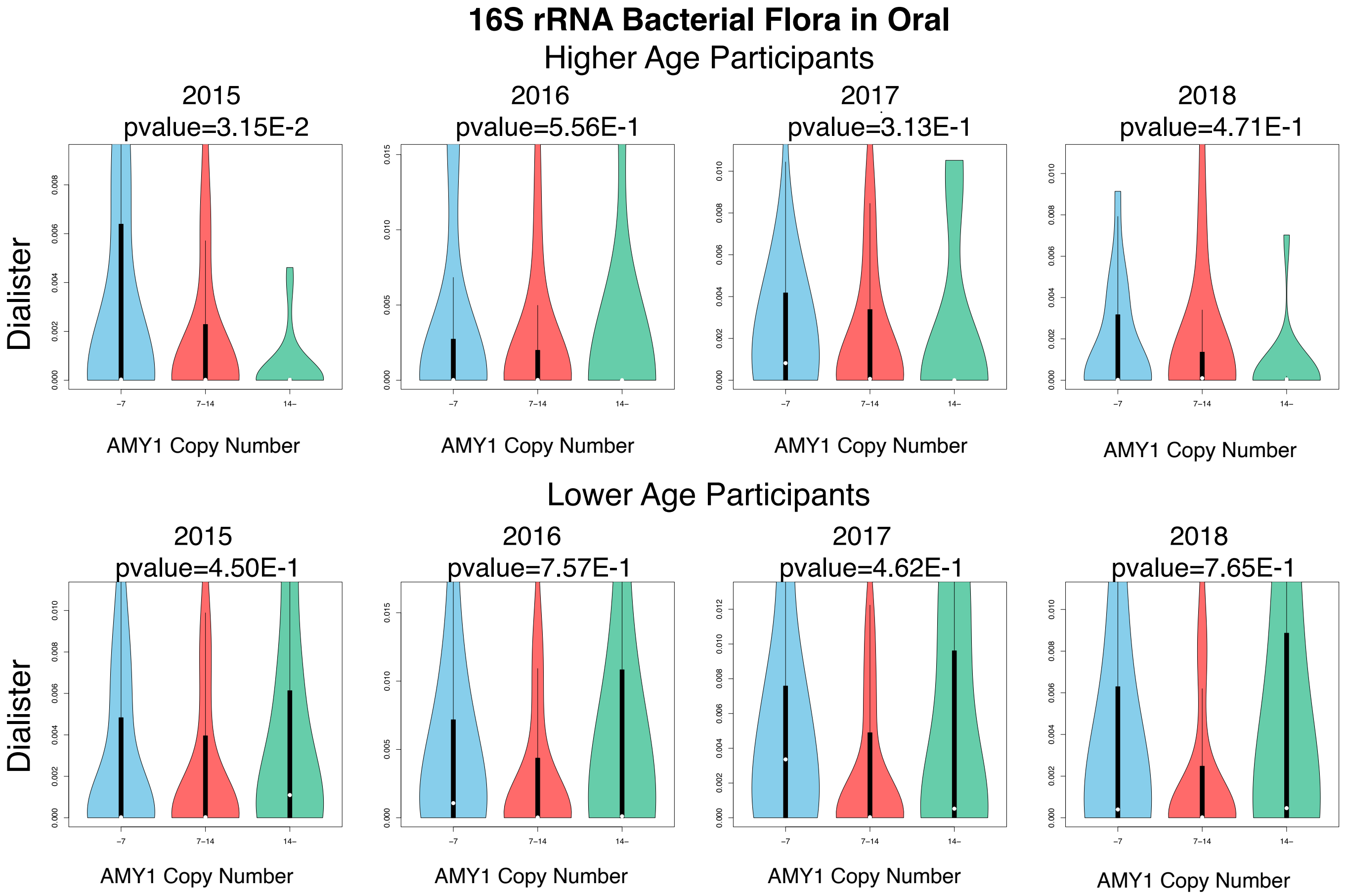
16S rRNA Bacterial Flora in Oral
Higher Age Participants
2015
pvalue=3.15E-2
2016
pvalue=5.56E-1
2017
pvalue=3.13E-1
2018
pvalue=4.71E-1
Dialister
AMY1 Copy Number
AMY1 Copy Number
AMY1 Copy Number
AMY1 Copy Number
Lower Age Participants
2015
pvalue=4.50E-1
2016
pvalue=7.57E-1
2017
pvalue=4.62E-1
2018
pvalue=7.65E-1
Dialister
AMY1 Copy Number
AMY1 Copy Number
AMY1 Copy Number
AMY1 Copy Number

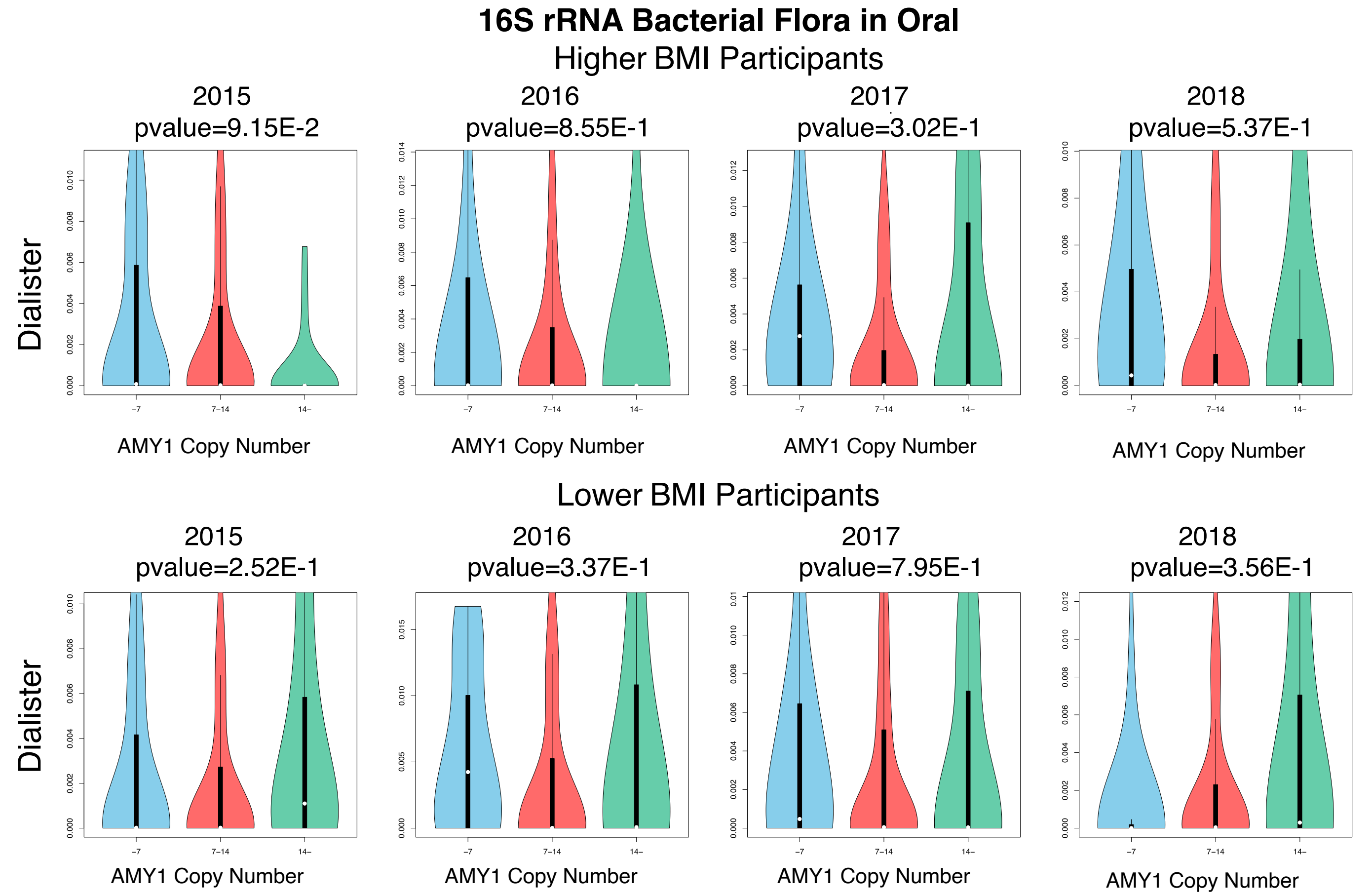
16S rRNA Bacterial Flora in Oral
Higher BMI Participants
2015
pvalue=9.15E-2
2016
pvalue=8.55E-1
2017
pvalue=3.02E-1
2018
pvalue=5.37E-1
Dialister
AMY1 Copy Number
−7
7−14
14−
Lower BMI Participants
2015
pvalue=2.52E-1
2016
pvalue=3.37E-1
2017
pvalue=7.95E-1
2018
pvalue=3.56E-1

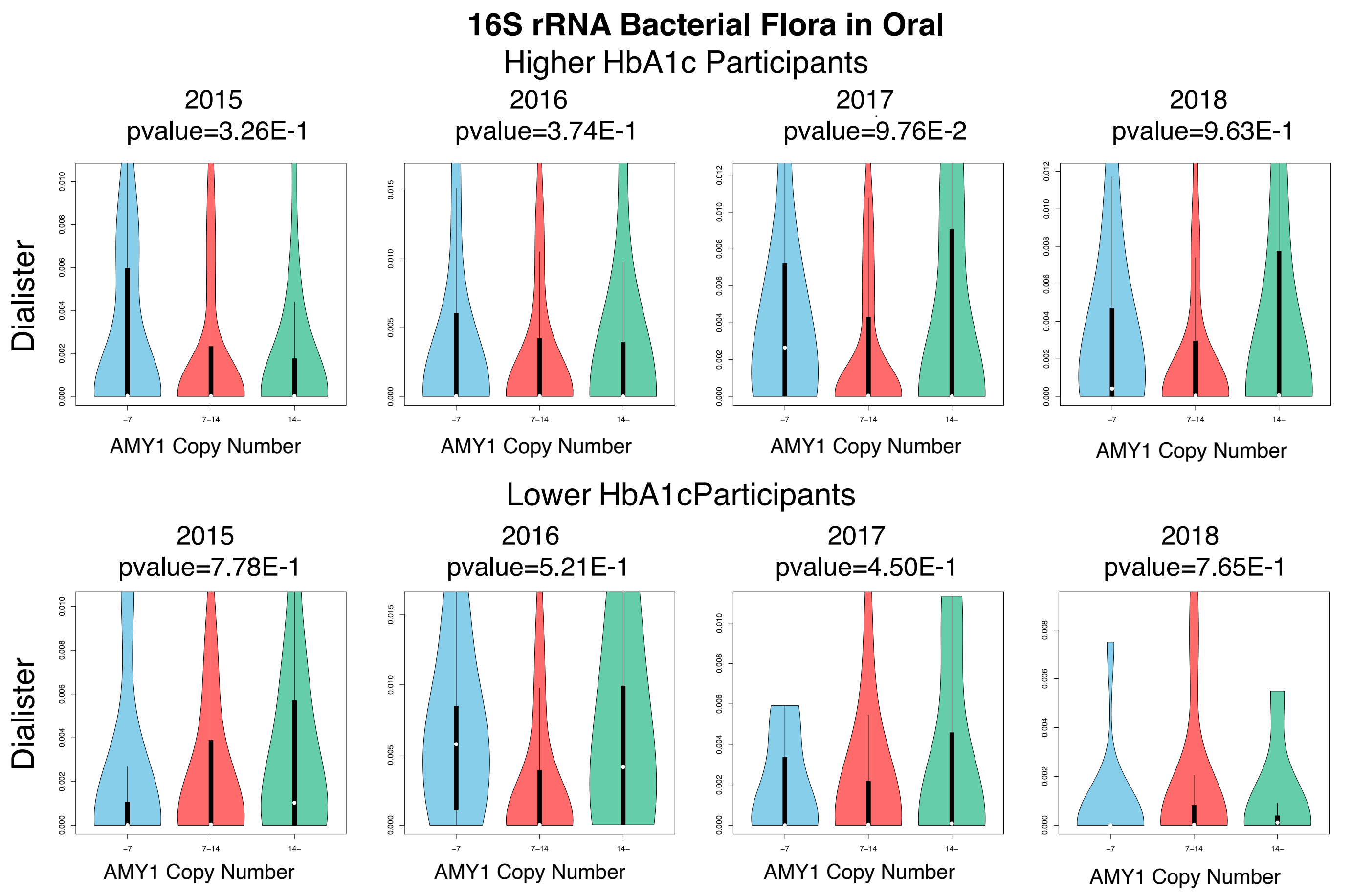

16S rRNA Bacterial Flora in Oral
Higher HbA1c Participants
2015
pvalue=3.26E-1
2016
pvalue=3.74E-1
2017
pvalue=9.76E-2
2018
pvalue=9.63E-1
Dialister
AMY1 Copy Number
Lower HbA1cParticipants
2015
pvalue=7.78E-1
2016
pvalue=5.21E-1
2017
pvalue=4.50E-1
2018
pvalue=7.65E-1
Dialister
AMY1 Copy Number

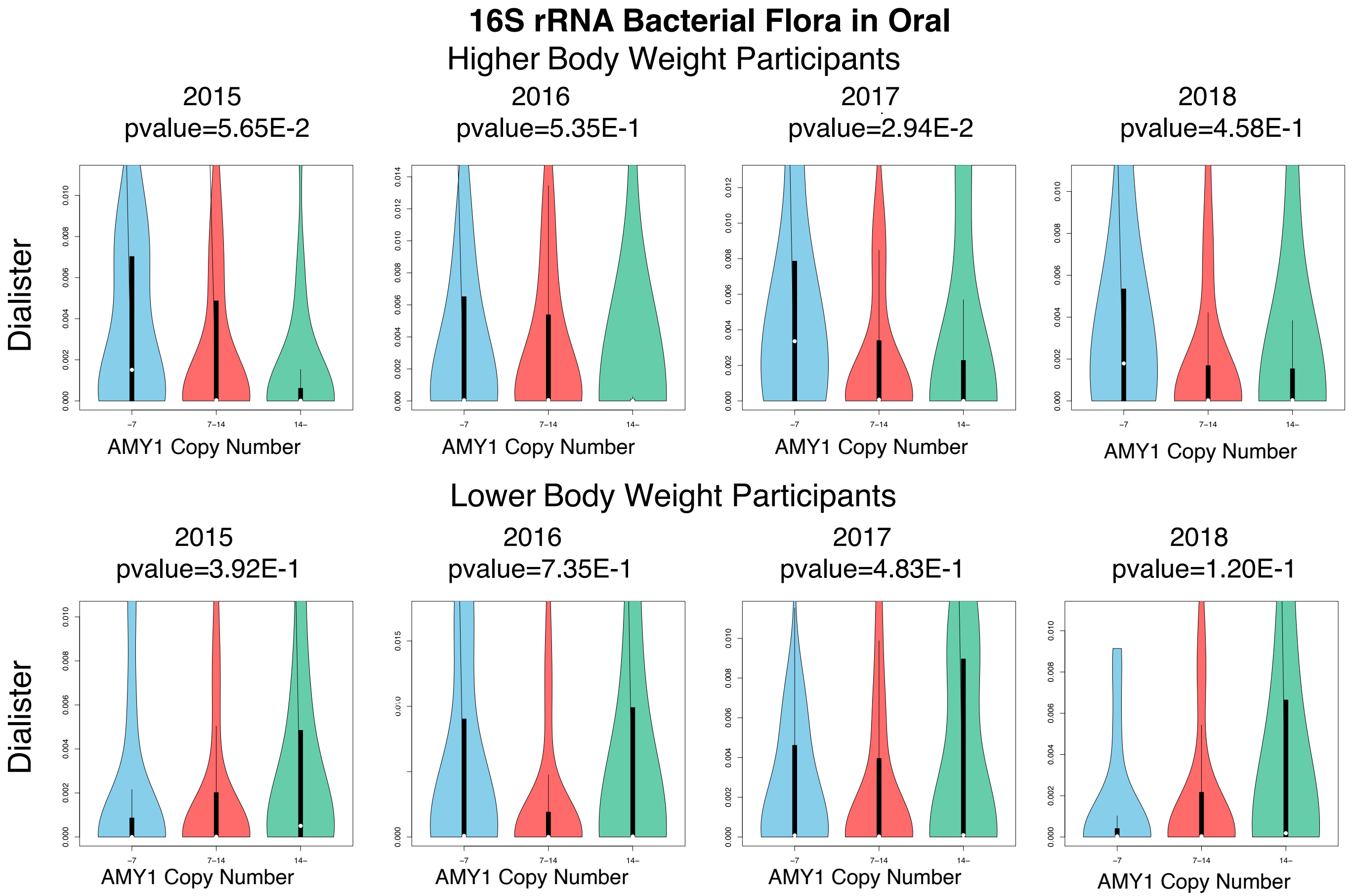
16S rRNA Bacterial Flora in Oral
Higher Body Weight Participants
2015
pvalue=5.65E-2
2016
pvalue=5.35E-1
2017
pvalue=2.94E-2
2018
pvalue=4.58E-1
Dialister
AMY1 Copy Number
-7
7-14
14-
Lower Body Weight Participants
2015
pvalue=3.92E-1
2016
pvalue=7.35E-1
2017
pvalue=4.83E-1
2018
pvalue=1.20E-1
Dialister
AMY1 Copy Number

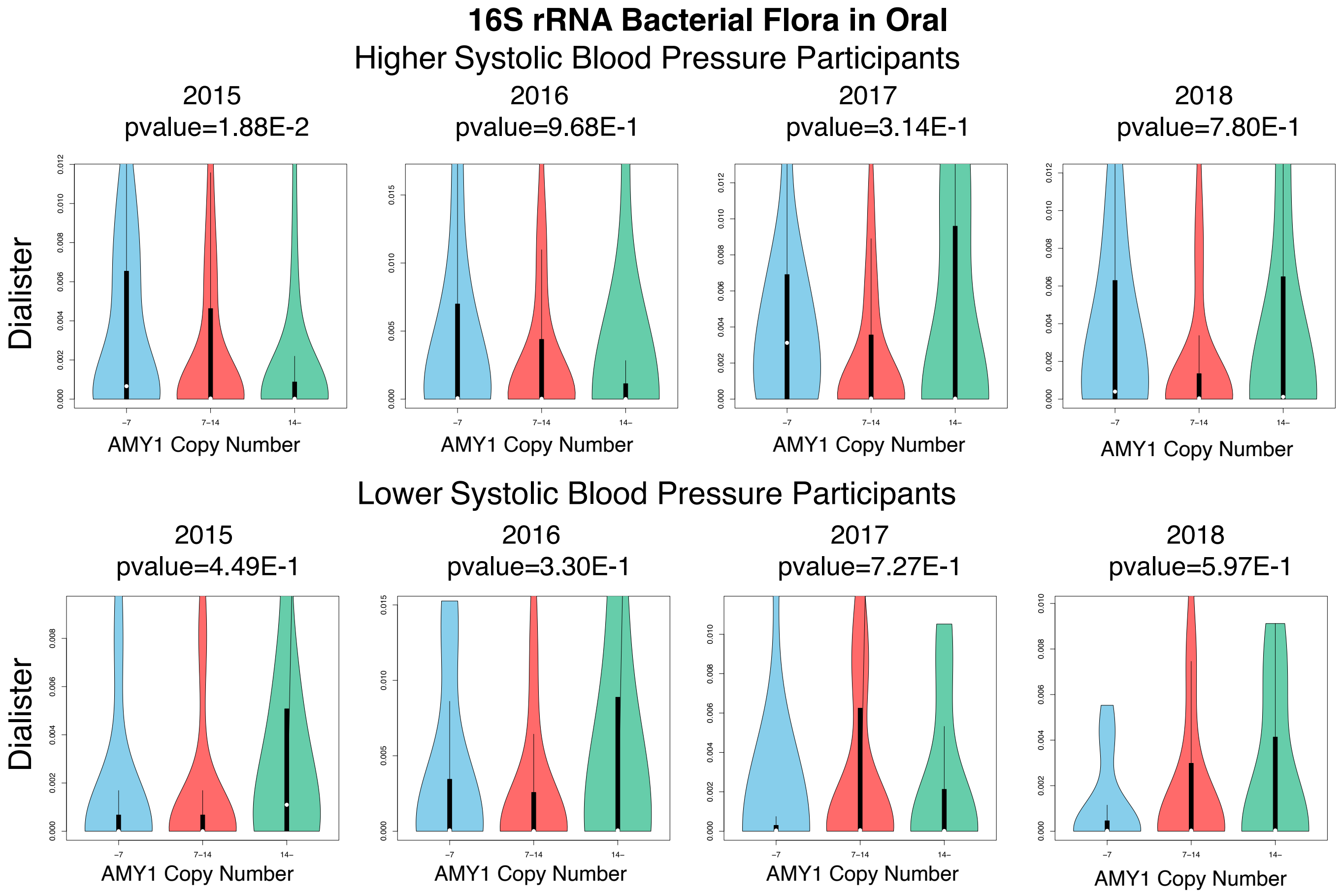
16S rRNA Bacterial Flora in Oral
Higher Systolic Blood Pressure Participants
2015
pvalue=1.88E-2
2016
pvalue=9.68E-1
2017
pvalue=3.14E-1
2018
pvalue=7.80E-1
Dialister
AMY1 Copy Number
–7
7–14
14–
Lower Systolic Blood Pressure Participants
2015
pvalue=4.49E-1
2016
pvalue=3.30E-1
2017
pvalue=7.27E-1
2018
pvalue=5.97E-1
Dialister
AMY1 Copy Number

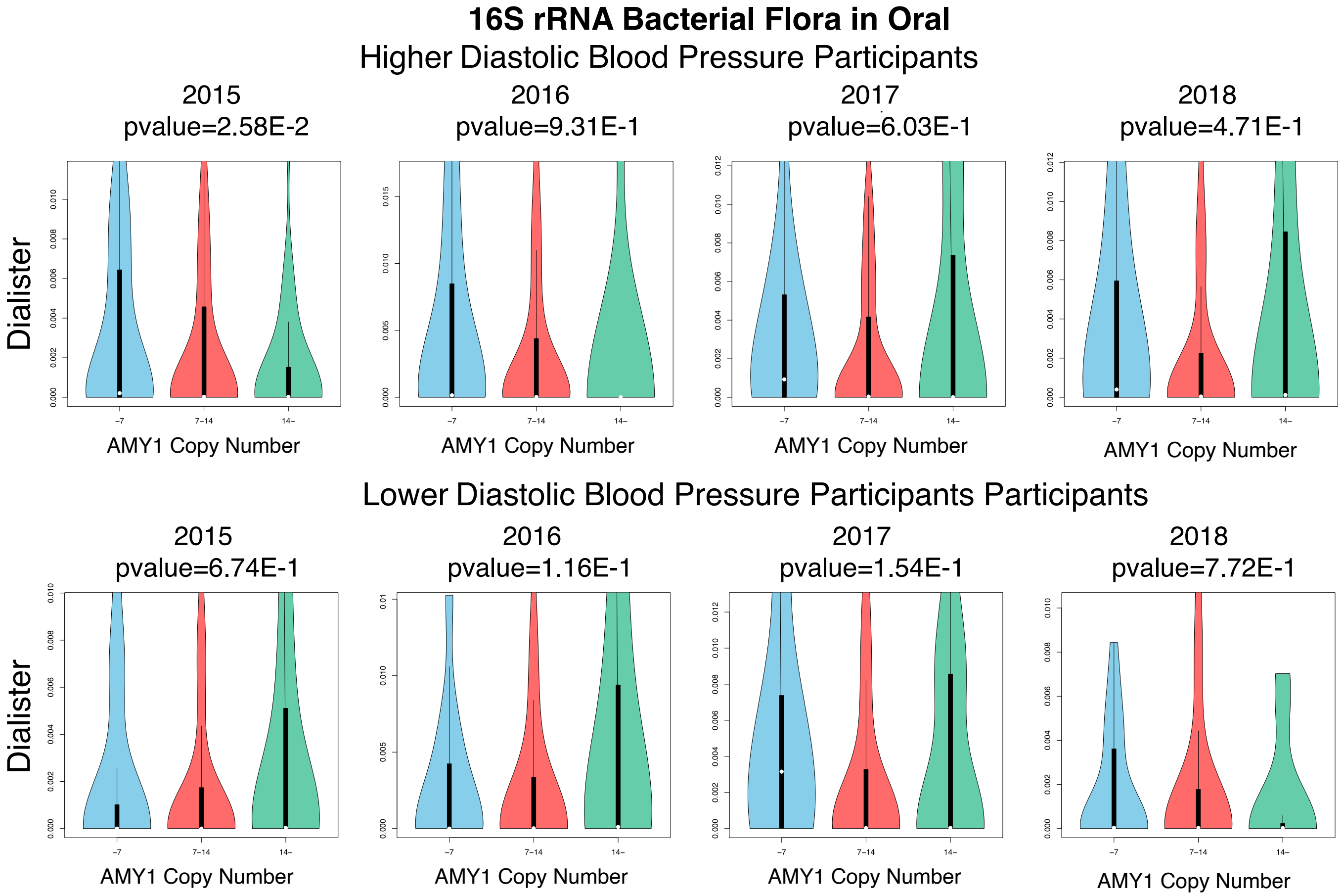

16S rRNA Bacterial Flora in Oral
Higher Diastolic Blood Pressure Participants
2015
pvalue=2.58E-2
2016
pvalue=9.31E-1
2017
pvalue=6.03E-1
2018
pvalue=4.71E-1
Dialister
AMY1 Copy Number
Lower Diastolic Blood Pressure Participants Participants
2015
pvalue=6.74E-1
2016
pvalue=1.16E-1
2017
pvalue=1.54E-1
2018
pvalue=7.72E-1
Dialister
AMY1 Copy Number

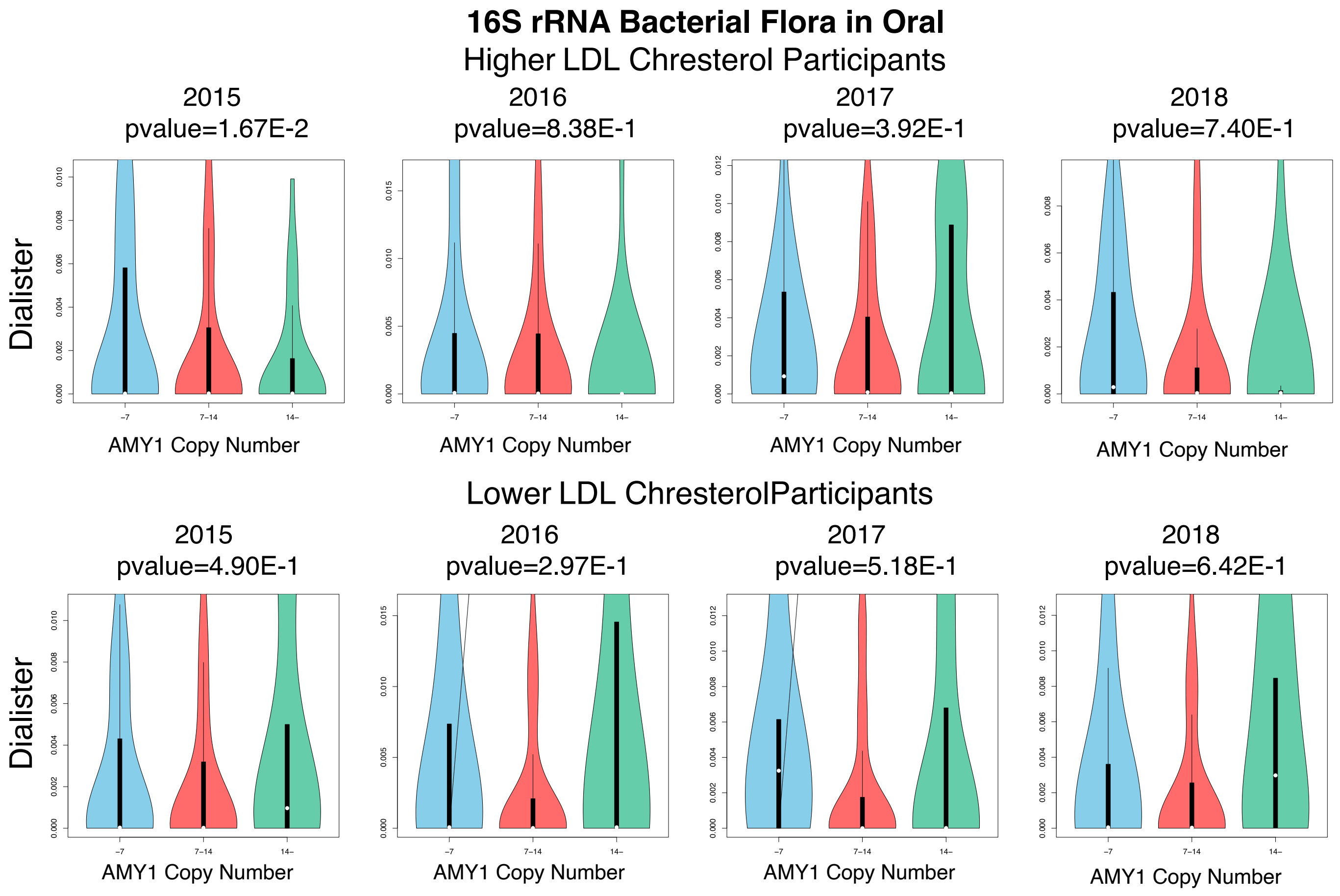
16S rRNA Bacterial Flora in Oral
Higher LDL Chresterol Participants
2015
pvalue=1.67E-2
2016
pvalue=8.38E-1
2017
pvalue=3.92E-1
2018
pvalue=7.40E-1
Dialister
AMY1 Copy Number
-7
7-14
14-
Lower LDL ChresterolParticipants
2015
pvalue=4.90E-1
2016
pvalue=2.97E-1
2017
pvalue=5.18E-1
2018
pvalue=6.42E-1
Dialister
AMY1 Copy Number

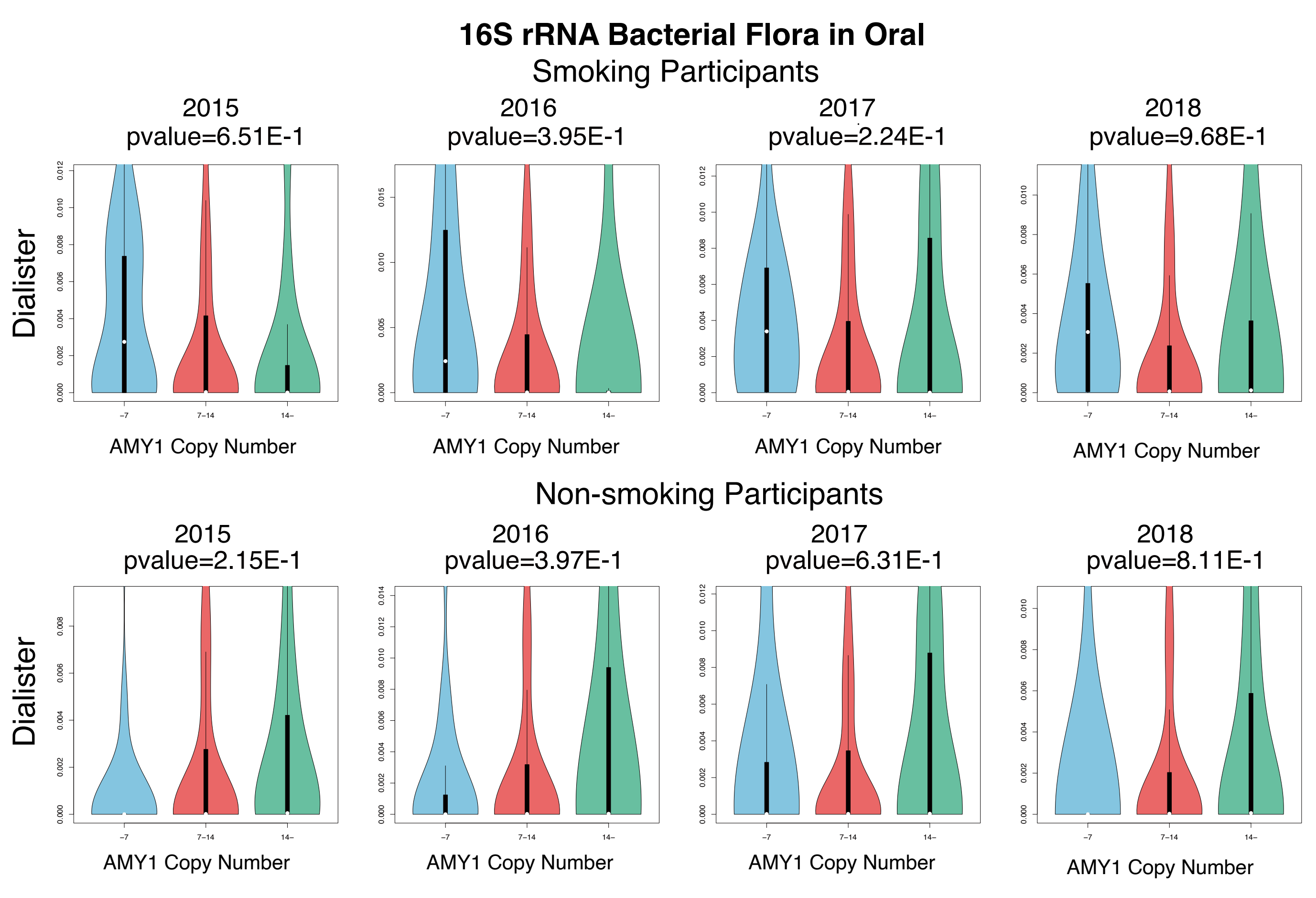
16S rRNA Bacterial Flora in Oral
Smoking Participants
2015
pvalue=6.51E-1
2016
pvalue=3.95E-1
2017
pvalue=2.24E-1
2018
pvalue=9.68E-1
Dialister
−7
7−14
14−
AMY1 Copy Number
Non-smoking Participants
2015
pvalue=2.15E-1
2016
pvalue=3.97E-1
2017
pvalue=6.31E-1
2018
pvalue=8.11E-1
Dialister
AMY1 Copy Number

16S rRNA Bacterial Flora in Oral

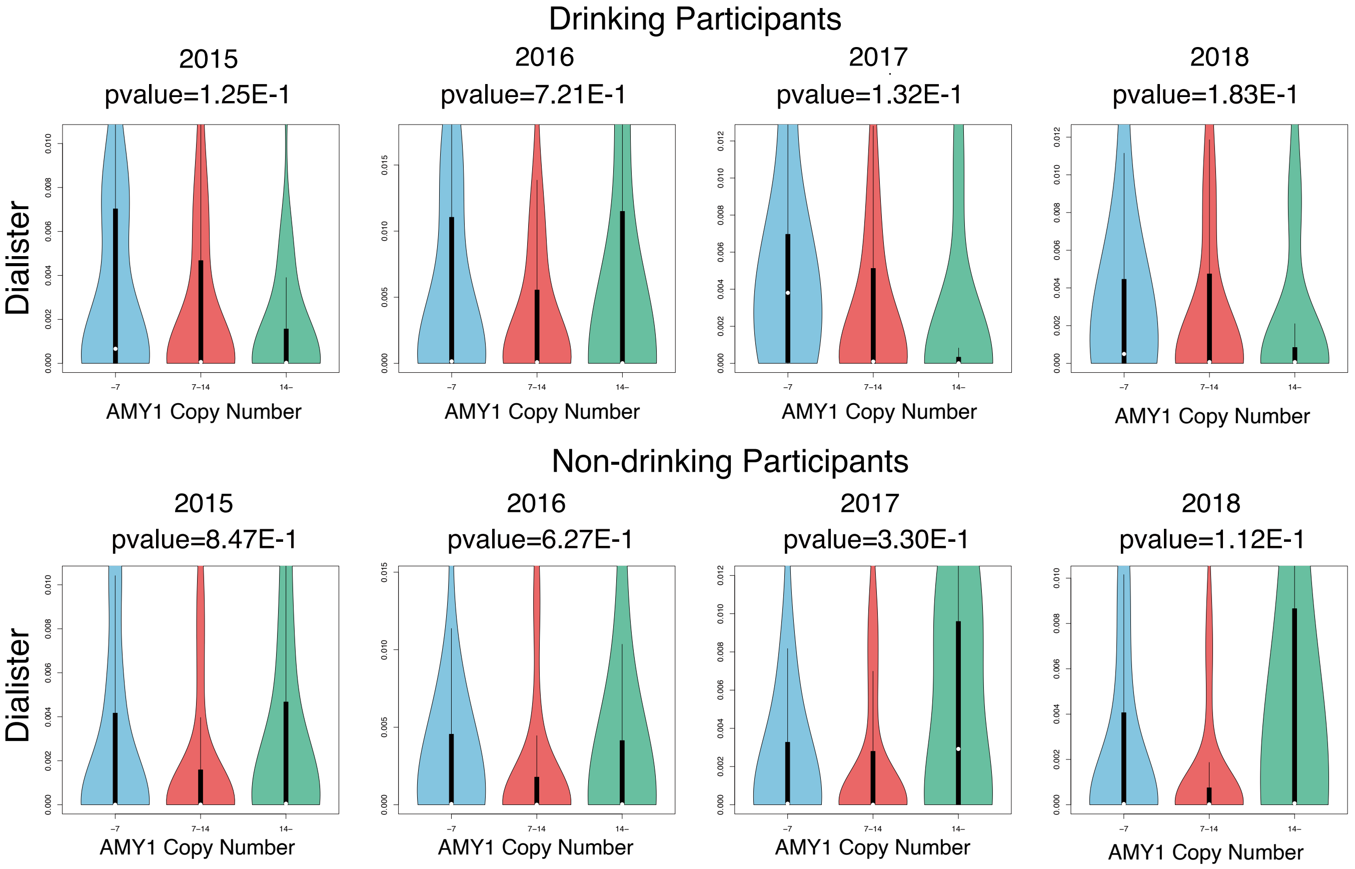

Supplement: Supplementary file 2 — Supplementary Information 2. [file 41598_2022_11730_MOESM2_ESM.pdf]
