# Supplementary material for: Impact of salivary and pancreatic amylase gene copy numbers on diabetes, obesity, and functional profiles of microbiome in Northern Japanese population
Source: Sci Rep. 2022 May 10;12:7628. doi: 10.1038/s41598-022-11730-7 (PMC9090785; doi:10.1038/s41598-022-11730-7)
Supplement: Supplementary file 4 — Supplementary Information 4. [file 41598_2022_11730_MOESM4_ESM.pdf]

Oral Microbiome (genus, p-value)											
Male and Female				Male				Female			
AMY1A		AMY2A		AMY1A		AMY2A		AMY1A		AMY2A	
apnocytophag	1.38492E-06	Alloscardovia	0.00252246	Bacteroides	0.00884817	Mycoplasma	0.00537596	Capnocytoph	9.7757E-07	Howardella	0.00122281
Lautropia	0.002875591	Desulfobulbu	0.00767486	Lautropia	0.01163532	Schwartzia	0.01497963	Fusobacteriu	0.00057786	Shuttleworth	0.00617352
Romboutsia	0.004980457	Treponema	0.0258245	Capnocytoph	0.01863692	Tannerella	0.01531352	Parvimonas	0.00645228	Peptoniphilus	0.01131323
Catonella	0.009130838	Achromobact	0.04165186	Catonella	0.01973323	Treponema	0.01586972	Campylobact	0.0128947	Alloscardovia	0.01399269
Saccharibact	0.013648724	Raoultella	0.05813924	Filifactor	0.03337993	Neisseria	0.01607126	Romboutsia	0.01376227	Cryptobacter	0.02490859
Fusobacteriu	0.017850754	Schwartzia	0.06380201	Enterococcus	0.034455	Peptoniphilus	0.02106509	Solobacteriu	0.01485281	Granulicatella	0.0378569
Peptostrepto	0.03286158	Pyramidobac	0.07264064	Escherichia/S	0.03862957	Scardovia	0.03884765	Rothia	0.02025476	Lautropia	0.04181737
Enterococcus	0.043417378	Escherichia/S	0.08075315	Deinococcus	0.04597273	Raoultella	0.03991252	Blautia	0.02662666	Roseburia	0.04285195
Campylobact	0.043509591	Propionibacte	0.08388968	Slackia	0.05350477	Comamonas	0.04269764	Cryptobacter	0.02893532	Niveispirillum	0.04341788
Solobacteriu	0.048392175	Phascolarcto	0.08444056	Peptostrepto	0.05612149	Filifactor	0.04531431	Mogibacteriu	0.03141558	Catonella	0.04462881

Gut Microbiome (genus, p-value)											
Male and Female				Male				Female			
AMY1A		AMY2A		AMY1A		AMY2A		AMY1A		AMY2A	
Megasphaera	0.001487438	Anaerostipes	0.0011348	Dialister	1.022E-06	Bifidobacteri	0.00167491	Dialister	6.0041E-06	Flavonifracto	3.4493E-05
Faecalibacte	0.001739769	Flavonifracto	0.00541643	Megasphaera	2.9204E-05	Gemella	0.0027667	Faecalibacte	0.00010316	Dialister	0.00575892
Flavonifracto	0.007713141	Gemella	0.00802741	Bifidobacteri	7.5115E-05	Methanobrev	0.00330896	Megamonas	0.00018452	Barnesiella	0.00604753
Coprococcus	0.010056927	Coprobacter	0.00857085	Veillonella	0.00034992	Stomatobacu	0.00924707	Flavonifracto	0.00036173	Lachnospirac	0.00629187
Pediococcus	0.010693062	Stomatobacu	0.00860853	Holdemanella	0.00125158	Anaerostipes	0.01421471	Alloprevotella	0.00283019	Enterococcus	0.00659771
Christensene	0.014092088	Enterococcus	0.00953113	Acidaminoco	0.00259936	Coprobacter	0.01748766	Stomatobacu	0.005165	Bilophila	0.00714666
Ruminococcu	0.014284955	Morganella	0.02101648	Prevotella	0.0107398	Dorea	0.01955454	Veillonella	0.00579802	Succinivibrio	0.0082315
Turicibacter	0.025007697	Dialister	0.02249705	Coprococcus	0.01876237	Buttiauxella	0.02320624	Eggerthella	0.01130972	Acidaminoco	0.01515036
Eggerthella	0.039521963	Bilophila	0.02732775	Olsenella	0.02155837	Megamonas	0.02544681	Clostridium X	0.01850992	Anaerofustis	0.02157995
Prevotella	0.042400996	Ruminococcu	0.0294968	Pediococcus	0.02222906	Alloscardovia	0.02775844	Romboutsia	0.02252999	Anaerostipes	0.02568245
